# Cost-benefit of a gamification-based intervention (ERES) in type 2 diabetes: Evaluating impacts on healthcare utilization and patient activation

**DOI:** 10.1192/j.eurpsy.2026.11678

**Published:** 2026-07-31

**Authors:** M. Sharifi, C. A. Lewis, M. Firoozi, S. Khani, P. Afkari

**Affiliations:** 1 Psychology, Islamic Azad University, Bandargaz Branch, Bandargaz, Iran, Islamic Republic Of; 2 Psychology, Leeds Trinity University, Leeds, United Kingdom; 3 Psychology, University of Tehran; 4 Psychology, Payam-e-noor University, Tehran, Iran, Islamic Republic Of

## Abstract

**Introduction:**

Type 2 diabetes (T2DM) imposes substantial economic burdens on healthcare systems.

**Objectives:**

This study aimed to evaluate the cost-benefit of a gamification-based intervention delivered through the ERES mobile application by examining its impact on healthcare utilization patterns and patient activation among Iranian women with T2DM.

**Methods:**

A quasi-experimental cost-benefit analysis was conducted with a pretest-posttest control group design. A purposive sample of 98 Iranian women with T2DM was randomly assigned to an intervention group (n = 49) that received eight 90-minute sessions of the ERES-based gamification intervention or a control group (n = 49) that received routine care. Data were collected using the Patient Activation Measure (PAM-13), healthcare utilization records (emergency visits, hospitalizations, outpatient consultations), and cost documentation. Analysis employed multivariate analysis of variance with repeated measures and cost-benefit calculation

**Results:**

Baseline analysis showed no significant differences between groups in activation or healthcare utilization (p > .05). Post-intervention results demonstrated significantly greater improvement in patient activation scores in the intervention group compared to controls (p < .01). More notably, the intervention group showed a 42% reduction in diabetes-related emergency visits and a 31% reduction in hospitalizations compared to the control group. Cost-benefit analysis revealed a 2.8:1 return on investment, indicating that for every $1 invested in the intervention, $2.80 was saved in avoided healthcare costs.

**Conclusions:**

The ERES gamification intervention not only enhanced patient activation but also significantly reduced healthcare utilization and generated substantial cost savings. These findings position digital gamification strategies as both clinically effective and economically advantageous for T2DM management. Healthcare policymakers and insurers should consider implementing such interventions to reduce the economic burden of diabetes while improving patient outcomes

**Disclosure of Interest:**

None Declared

